# Workplace health and safety under climate stress in Sri Lankan apparel SMEs

**DOI:** 10.1186/s12889-026-26344-1

**Published:** 2026-01-20

**Authors:** Devathanthrige Janaka Chamara Harshana Senadeera, Abu Sadat Muhammad Sayem, Walter Leal Filho, Grace Farhat, Haruna Musa Moda

**Affiliations:** 1 Faculty of Health and Education, Manchester Metropolitan University, Manchester, United Kingdom; 2 School of Design and Creative Arts, Loughborough University, Loughborough, United Kingdom; 3 Department of Natural Sciences, Manchester Metropolitan University, Manchester, United Kingdom; 4 Research and Transfer Centre, Sustainable Development & Climate Change Management “(FTZ-Hamburg University of Applied Sciences, Ulmenliet 20, Hamburg, 21033 Germany; 5 Department of Environmental Health and Safety, University of Doha for Science and Technology, Doha, Qatar

**Keywords:** Climate change impacts, Workplace health and safety, Sri Lankan apparel industry, Small and medium manufacturing companies

## Abstract

Climate change poses escalating risks to labor-intensive industries in South Asia. This mixed‑methods study assessed how climate‑related hazards affect the health, safety and productivity of workers in Sri Lankan small‑ and medium‑scale apparel manufacturing companies (SMAMCs). A survey of 384 employees in Biyagama and Katunayake Export Processing Zones captured quantitative data on exposure to excessive heat, flooding, indoor air pollution and mosquito‑borne diseases, and elicited qualitative accounts of workplace experiences. Heat waves and high humidity were the most pervasive stressors: 81.25% (95% CI; 77.3 to 85.2) of respondents reported heat stress, with headaches, dehydration, and diminished concentration frequently linked to needle‑prick injuries. Flood events damaged infrastructure and heightened respiratory, gastrointestinal and dermatological illnesses, while inadequate ventilation compounded air‑quality problems; 95% (95% CI; 92.4 to 96.8) of workers complained of persistent coughing. Dengue incidence over the preceding five years reached 10.68% (95% CI; 8.0 to 14.2), reflecting expanding vector habitats. Chi‑square analysis confirmed statistically significant associations between each hazard and adverse health outcomes. The study underscores the need for integrated adaptation measures, improved ventilation, low‑cost cooling, drainage upgrades and systematic vector control, supported by enforceable regulations and targeted financial assistance. Enhancing climate resilience in SMAMCs is essential for safeguarding worker wellbeing and sustaining Sri Lanka’s export competitiveness, while contributing to global goals on decent work and climate action targets.

**Supplementary Information:**

The online version contains supplementary material available at 10.1186/s12889-026-26344-1.

## Key Highlights

1. Mandate climate-resilient workplace health and safety standards for heat, flooding, indoor air quality and vector risks in SMAMCs, with inspectorate enforcement and auditable compliance logs.
2. Provide targeted grants and technical assistance so that resource constrained small and medium scale enterprises can retrofit ventilation, passive cooling and flood proofing without eroding competitiveness.
3. Require real time monitoring of temperature, heat index and airborne particulates on production floors, alongside staggered shifts, task rotation and scheduled hydration and rest breaks to reduce dehydration, cognitive fatigue and respiratory illness.
4. Integrate factory rules with public vector control and municipal environmental health services to curb dengue in export processing zones, reduce absenteeism and protect continuity of operations, thereby supporting Sustainable Development Goal 8 and Sustainable Development Goal 13.
5. Embed worker participation and continuous training through toolbox talks, incident reporting and corrective action tracking to keep controls effective over time.
6. Include climate risk assessment in factory licenses and annual audits, with heat and flood action plans, maintenance of drainage and sumps, and verified emergency egress testing.
7. Prioritise low-cost engineering controls such as reflective roofing, shading, cross ventilation, local exhaust and raised electrical isolation above flood lines as primary measures, with personal protective equipment used only as residual protection.
8. Establish performance indicators for heat illness, dehydration events, respiratory complaints and dengue related absence, and publish aggregated results to drive continuous improvement and benchmarking.

**Supplementary Information:**

The online version contains supplementary material available at 10.1186/s12889-026-26344-1.

## Introduction

The global apparel industry plays a critical role in economic development and employment generation, particularly in developing countries such as Bangladesh, Sri Lanka, Vietnam, and Ethiopia, where it accounts for a substantial share of exports and labour participation [1, 2]. The apparel sector, given its highly labor-intensive nature, not only acts as an engine of economic growth but also supports millions of livelihoods [3], underpinning broader socio-economic stability in these nations. Consequently, the sustainability and resilience of this sector are paramount, especially against the backdrop of escalating environmental threats.

Nevertheless, despite its considerable economic importance, the global apparel industry faces increasing exposure to climate-induced hazards such as floods, heatwaves, and droughts, which disrupt operations, damage infrastructure, and threaten worker health and productivity [4]. The vulnerability of the apparel industry to climate hazards emerges from multiple factors, including geographical susceptibility, inadequate infrastructure, limited resources allocated to climate adaptation, and systemic weaknesses in policy implementation [3, 4]. These factors intensify risks, especially in countries reliant on this sector for economic stability and employment continuity.

Apparel production hubs across the Global South face converging climate risks that affect worker health and factory continuity. In Bangladesh, repeated flooding and rising wet-bulb temperatures have disrupted production and heightened heat-related risks for garment workers [5, 6]. In India, factory-level evidence links higher temperatures to measurable output losses in manufacturing, and urban flooding has disrupted major textile clusters such as Surat; worker-centered research from Bengaluru documents heat-related health burdens among garment workers [7–9]. In Cambodia, factory-floor research shows that higher ambient temperatures are associated with worse symptoms and lower perceived productivity during hotter months [10]. In Vietnam, assessments underline the vulnerability of major apparel centres to extreme heat and flooding and outline facility-level resilience measures [6]. Evidence from East Africa likewise documents substantial heat-exposure symptoms among industrial workers, underscoring the generalisability of thermal risk across low-cost manufacturing environments [11]. Collectively, these examples indicate that climate-related heat and flood hazards are widespread across apparel supply chains and reinforce the need for integrated, hierarchy-of-controls approaches alongside national policy alignment [10, 12].

The implications of these risks are particularly profound in labor-intensive environments, where workplace health and safety are intrinsically linked to environmental stability. The apparel industry’s workforce is predominantly concentrated in developing regions, where workers often experience precarious working conditions compounded by limited access to adequate health and safety provisions [13]. Factories, particularly small- and medium-scale enterprises, frequently lack the structural and operational capacity to manage climate-induced occupational hazards effectively [10, 14], thereby substantially compromising worker well-being.

In South Asia, apparel production countries such as Bangladesh, India, and Sri Lanka are especially reliant on this sector for economic growth, foreign exchange earnings, and employment generation. Bangladesh, for instance, exemplifies the critical role apparel exports play, comprising approximately 80% of its total exports [1, 2]. India and Sri Lanka similarly depend substantially on apparel manufacturing to drive economic growth and employment, yet all three countries grapple with persistent issues concerning workplace safety standards. Despite extensive international attention following high-profile disasters, such as Bangladesh’s Rana Plaza collapse, many factories across the region still face inadequate fire prevention systems, poor ventilation, and deficient emergency protocols [13, 15]. These shortcomings are particularly pronounced within small- and medium-scale enterprises, which typically possess limited financial and technical capacities, thus exacerbating their susceptibility to climate-induced hazards [4]. Smaller apparel factories, notably those operating in Bangladesh, frequently experience critical gaps in structural integrity, emergency exits, and air-quality management, placing workers at continual risk of occupational hazards [16, 17]. Enforcement of safety standards across the apparel sector remains inconsistent, influenced by governance challenges such as corruption, regulatory fragmentation, and inadequate oversight, disproportionately affecting smaller and subcontracted facilities [16, 17]. Addressing climate-induced health and safety concerns within smaller apparel manufacturing firms therefore demands focused policy intervention and sustained institutional support.

Several climate-induced hazards pose escalating threats to apparel-producing regions. Excessive heat, a widely documented hazard, contributes to worker fatigue, dehydration, cognitive impairment, and substantial productivity losses, particularly in factories lacking adequate thermal management systems [7, 14, 18]. For example, research from Bangladesh and India highlights how increasing indoor temperatures directly correlate with higher incidents of heat stress among apparel workers, reducing both worker well-being and operational efficiency [9, 19]. These studies underscore the need for industry-specific adaptation measures to mitigate heat-related health risks.

Poor air quality within enclosed factory spaces constitutes another important occupational hazard, exacerbated by climate variability [20]. Emissions from textile dyes, chemical processing, and insufficient ventilation systems have been associated with chronic respiratory diseases, cardiovascular ailments, and reduced overall worker health [21]. While large-scale factories might employ sophisticated ventilation solutions, smaller factories frequently rely on rudimentary ventilation methods, which are insufficient to mitigate air pollutants effectively, especially during periods of elevated external temperatures or extreme weather conditions [13, 16].

Vector-borne diseases such as dengue, malaria, and chikungunya represent an additional climate-induced hazard with substantial impacts on worker health in apparel-producing regions. Changing precipitation patterns, increasing temperatures, and inadequate drainage infrastructures intensify mosquito breeding, heightening the risk of outbreaks [22, 23]. Increased rainfall events and prolonged periods of stagnant water, particularly common during monsoonal seasons, create conducive conditions for disease transmission [24, 25]. These vector-borne health threats disproportionately affect regions with inadequate environmental health infrastructure, posing severe risks to the health and productivity of the apparel workforce in Sri Lanka and similar countries in the region [26].

### Climate change and the Sri Lankan apparel industry

Sri Lanka’s apparel industry underpins national exports and employment, generating c.40% of export value and over USD 5 billion annually, with more than 350,000 direct jobs and extensive indirect livelihoods, especially for women [27–29]. Climate change intensifies risks to operational continuity and worker wellbeing, particularly for small and medium-scale apparel manufacturing companies (SMAMCs), which often operate in ageing facilities with limited HVAC capacity and constrained resources for workplace health and safety, heightening exposure to climate-related hazards [13, 15, 16]. Projections indicate a mean temperature rise of 1.0–1.5 °C by 2050, alongside increasingly erratic monsoonal rainfall that drives more frequent floods and droughts, threatening infrastructure, supply chains, and worker health [30–32].

Impacts fall disproportionately on SMAMCs due to dense workspaces, restricted air movement, and limited risk-management capacity; by contrast, larger firms more often leverage international partnerships and finance to implement adaptive measures [13, 16, 21, 33]. Yet research and policy attention have largely centered on large, export-oriented factories, leaving SMAMCs comparatively overlooked an omission that undermines sector-wide resilience, worker safety, and international competitiveness [13, 16, 21, 33].

Addressing this gap, the present study examines climate-induced occupational hazards, health outcomes, and current safety practices in SMAMCs located in the Biyagama and Katunayake Export Processing Zones, identifying prevalent hazards, related health implications, and the adequacy of existing controls. The study seeks to inform targeted, climate-resilient workplace strategies aligned with global agendas specifically the United Nations Sustainable Development Goals 8 (Decent Work and Economic Growth) and 13 (Climate Action) and to support integration of climate considerations into workplace health and safety governance for sustainable, safe, and resilient apparel workplaces in Sri Lanka [13, 16, 21, 27, 33].

## Materials and methods

This study was conducted within the Sri Lankan apparel manufacturing sector, focusing on small and medium-scale apparel manufacturing companies (SMAMCs) located in the Biyagama and Katunayake Free Trade Zones. These zones collectively host around 200 apparel companies and employ approximately 100,000 workers, forming the basis of the study population. Prior to commencing fieldwork, organisational access and site permissions were secured from six participating factories. Within each factory, paper questionnaires and participant information sheets were randomly distributed among production workers, with participation strictly voluntary and based on informed consent. Although design features may introduce modest selection bias, voluntary on-site participation, English-language with ≥2-year tenure, consenting factories, and two-zone sampling, these constraints are unlikely to overturn core patterns [34, 35]. In total, 384 valid responses were obtained. Data collection was carried out over six months, from 4 October 2023 to 27 March 2024, allowing the study to capture seasonal and environmental variations relevant to climate-related workplace risks. 

A mixed-methods research design [36] was adopted to investigate how climate-induced hazards affect workplace health and safety in SMAMCs. The quantitative component comprised a structured survey designed and developed specifically for this study to assess workers’ exposure to climate-related hazards and associated health risks. To complement and deepen these findings, open-ended questions were integrated within the survey instrument, enabling the collection of qualitative responses for thematic analysis. This methodological integration allowed for a more comprehensive understanding of both measurable trends and individual perceptions.

The sample size of 384 was determined using the Krejcie and Morgan [37] table, ensuring a 95% confidence level and a 5% margin of error. Participants included a diverse range of roles such as team members, multi-skilled operators, assistant team leaders, team leaders, and sectional heads, capturing variation in workplace exposure and responsibilities. While gender was not a selection criterion, it is well established that over 80% of the Sri Lankan apparel workforce is female, with more than 300,000 women employed directly [28, 38]. Observations during site visits by the first author confirmed the predominance of women on production floors, aligning with national employment patterns [39]. The primary focus of this study was on the interaction between climate change and workplace health and safety, rather than gender-based analysis.

### Data collection methods

To assess current health and safety risks in the context of climate hazards, a pre-tested and pilot-validated questionnaire was used. The instrument was developed from a structured synthesis of international guidance and recent evidence [14, 21, 26, 40]. Core domains heat stress, flood disruption, vector-borne disease, air quality, water and sanitation, and management-system practices, were derived from the thesis literature review and translated into items. The instrument was reviewed by six academic experts in workplace health, safety and climate change, and a 10-participant pilot tested validity, clarity and response processes, prompting minor wording and instruction refinements. The study protocol, including the questionnaire, was reviewed and approved by Manchester Metropolitan University’s independent Research Ethics Committee prior to fieldwork (EthOS reference number: 45145). Reliability was measured using Kuder–Richardson Formula 20 (KR-20) on the full sample; all retained scales were ≥0.70 [41–43].

Participants completed a self-administered questionnaire distributed on site by participating apparel firms that had provided letters of intent permitting employee participation. Respondents completed the instrument independently and returned it anonymously to a locked submission box kept at each site for four weeks; only the researcher retained the key. The questionnaire was developed and administered in English. Inclusion criteria were employees aged ≥18 years with English-language and at least two years’ continuous experience in their current factory, ensuring sufficient exposure to operations and climatic variability. Exclusion criteria were employees aged <18 years without English Language and <2 years’ experience.

### Statistical and qualitative analysis techniques

The quantitative data were analysed using IBM SPSS version 29 (Armonk, New York, USA). Descriptive statistics were used to summarise demographic attributes and key safety-related variables. Inferential analysis was conducted using Pearson’s Chi-square test [44, 45] to identify associations between categorical variables such as job role, work experience, and perceived health risks. To assess internal reliability, Cronbach’s Alpha [46] was applied for Likert-type items, while the Kuder-Richardson Formula 20 (KR-20) was used for binary items, confirming the validity and consistency of the dataset.

**Fig. 1:**
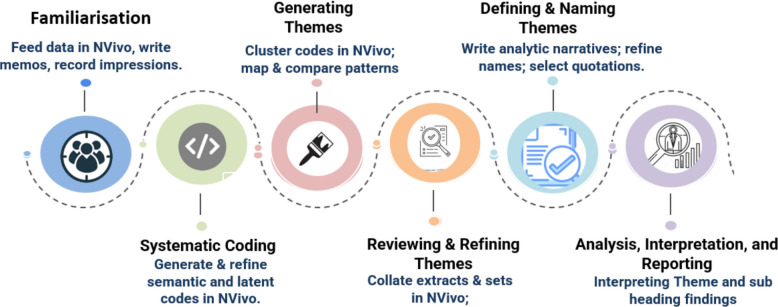
Thematic analysis process (source; [47, 48])

The qualitative responses from four open-ended survey questions were processed using NVivo version 15 (Denver, Colorado, USA) for thematic analysis (See Fig. 1). Coding was performed based on key phrases and recurrent terminology to identify dominant themes and sub-themes [47, 48]

This enabled the development of a thematic framework reflecting climate change-induced hazards. The integration of these qualitative insights with the quantitative findings provided a more nuanced understanding of how climate change is influencing occupational health and safety dynamics in Sri Lanka’s apparel manufacturing context.

## Results

The analysis quantified the impacts of four major climate-related hazards on employees in Sri Lankan SMAMCs. Adverse weather events such as floods, cyclones, landslides, lightning, and strong winds were reported by 80.73% (95% Confidence Interval [CI] 76.8 to 84.7) of respondents. Excessive heat impacted 81.25% (95% CI; 77.3 to 85.2), air pollution 44.53% (95% CI; 39.5 to 49.5), and mosquito-borne diseases 83.07% (95% CI; 79.3 to 86.8) (Table 1). Pearson chi-square tests confirmed the statistical significance of these impacts: adverse weather (χ^2^ = 28.380, *p <* 0.001), heat waves (χ^2^ = 29.790, *p <* 0.001), air pollution (χ^2^ = 36.050, *p <* 0.001), and mosquito-borne diseases (χ^2^ = 7.280, *p <* 0.007). The Phi values indicate weak to moderate associations: 0.272 (adverse weather), 0.279 (excessive heat), 0.306 (air pollution), and 0.138 (mosquito-borne diseases).

**Table 1:** Statistical significance of main climate change-induced natural hazards

Climate hazard	Percentage affected (%)	95% CI	Pearson chi-square	*P*-value	Phi value
Adverse Weather Conditions	80.73	76.8–84.7	28.38	*P <* 0.001	0.272
Excessive Heat	81.25	77.3–85.2	29.79	*P <* 0.001	0.279
Air Pollution	44.53	39.5–49.5	36.05	*P <* 0.001	0.306
Mosquito-borne Diseases	83.07	79.3–86.8	7.28	*P <* 0.007	0.138

For each hazard, participants reported the workplace risks and impacts summarised in Tables 1, 2, 3 and 4.

**Table 2:** Statistical significance of adverse weather-related health impacts

Health impact	Percentage affected (%)	95% CI	Pearson chi-square	*P*-value	Phi value
Respiratory Issues	75.52	71.2–79.8	5.63	*P <* 0.018	0.121
Asthma Symptoms	77.08	72.9–81.3	4.7	*P <* 0.003	0.111
Diarrhoea	47.4	42.4–52.4	6.81	*P <* 0.009	0.133
Sinus	73.7	69.3–78.1	15.82	*P <* 0.001	0.203
Throat Infections	81.25	77.3–85.2	5.58	*P <* 0.018	0.121
Stress	29.95	25.3–34.6	16.0	*P <* 0.001	0.204

**Table 3:** Statistical significance of excessive heat-induced risks

Risk type	Percentage affected (%)	95% CI	Chi-square	*P*-value	Phi value
Excessive Sweating	89.58	86.1–92.3	69.66	*P <* 0.001	0.426
Severe Headache	85.67	81.8–88.8	53.99	*P <* 0.001	0.375
Feeling Faintness	84.89	81.0–88.1	77.59	*P <* 0.001	0.450
Heat Rash	89.84	86.4–92.5	72.61	*P <* 0.001	0.553
Dehydration	80.46	76.2–84.1	43.23	*P <* 0.001	0.336
Reduced Focus	85.41	81.5–88.6	75.78	*P <* 0.001	0.444
Extreme Fatigue	84.89	81.0–88.1	75.32	*P <* 0.001	0.443
Workplace Hazards	60.15	55.2–64.9	53.2	*P <* 0.001	0.372

**Table 4:** Statistical significance of air pollution-related health impacts

Health impact	Percentage affected (%)	95% CI	Pearson chi-square	*P*-value	Phi value
Coughing	95.05	92.4–96.8	1.358	0.244	0.0595
Shortness of Breath	70.31	65.6–74.7	21.779	*P <* 0.001	0.238
Respiratory Issues Symptoms	94.79	92.1–96.6	3.258	0.071	0.092
Allergies	94.01	91.2–96.0	0.941	0.332	0.050
Eye Diseases	70.57	65.8–74.9	18.95	*P <* 0.001	0.222

### Adverse weather conditions

Adverse weather conditions were linked to several health issues among SMAMCs employees. Reported symptoms included respiratory problems 75.52% (95% CI; 71.2 to 79.8), asthma 77.08% (95% CI; 72.9 to 81.3), diarrhoea 47.40% (95% CI; 42.4 to 52.4), sinus issues 73.70% (95% CI; 69.3 to 78.1), and throat infections 81.25% (95% CI; 77.3 to 85.2). Mental health effects included stress at 29.95% (95% CI; 25.3 to 34.6). Pearson chi-square tests confirmed statistically substantial associations (Table 2).

Analysis of qualitative responses identified two primary themes relating to the impacts of flooding on workplace health and safety in Sri Lanka’s SMAMCs:

#### Physical safety risks

Workers frequently reported experiencing physical injuries due to unsafe conditions exacerbated by flooding events. Commonly described hazards included slippery communal areas, structural instability, and falling debris from compromised building infrastructure. One participant highlighted the immediate risk associated with wet floors, stating, “I fell near the canteen area after the rain, the floor was wet and no sign.” Another respondent detailed structural failure, noting, “The wall behind the packing section cracked and part fell during the last flood.” These examples underscore how inadequate maintenance and poor structural integrity significantly elevate physical injury risks during adverse weather conditions.

#### Flood-related health issues

Participants also described a range of health complications arising from flood exposure, notably sore eyes, ringworm, hepatitis, respiratory infections, and flu-like symptoms. Workers commonly associated these illnesses with contact with contaminated floodwater and prolonged exposure to cold and damp conditions inside factories. One respondent noted, “When it rains, we walk through the flood to transport, later we get throat pain and fever.” Another participant highlighted respiratory health risks linked to poor infrastructure, stating, “Windows are not properly fixed, mist comes, we get breathing difficulties while raining, later we get sick.” Gastrointestinal issues were also prevalent, particularly during the monsoon season, as reflected by another worker's comment: “Most employees experience loose motion issues after the flood season due to well water usage.”

### Excessive heat

Table 3 illustrates the substantial health risks posed by excessive heat in Sri Lanka’s SMAMCs, with most participants reporting adverse symptoms: 89.58% (95% CI; 86.1 to 92.3) reported excessive sweating, 85.67% (95% CI; 81.8 to 88.8) reported severe headaches, 84.89% (95% CI; 81.0 to 88.1) felt faint, 89.84% (95% CI; 86.4 to 92.5) developed heat rashes, and 80.46% (95% CI; 76.2 to 84.1) reported dehydration. Additionally, 85.41% (95% CI; 81.5 to 88.6) reported reduced focus, 84.89% (95% CI; 81.0 to 88.1) reported extreme fatigue, and 60.15% (95% CI; 55.2 to 64.9) encountered workplace hazards. Chi-square tests confirmed statistically substantial associations across all symptoms (*p <* 0.001), with Phi values ranging from 0.336 to 0.553, indicating moderate to strong associations.

Qualitative responses, participants described multiple physical health issues directly attributed to working in excessively hot conditions. Commonly reported symptoms included extreme fatigue affecting nearly two-third of the polluation, reduced physical strength, persistent tiredness, and energy depletion during work shifts. Workers frequently mentioned physical ailments such as sore eyes, cuts and grazes, insomnia, and a notable loss of appetite. One participant specifically stated, “Sore eyes are spreading during the hot season in the factory.” Additionally, several workers highlighted dermatological problems, describing episodes of skin irritation, blisters, and rashes exacerbated by heat exposure. As one worker explained, “I keep scratching my calf on hot days, and then the rash turns into blisters.”

Workers further reported that fatigue and reduced concentration due to heat significantly increased the risk of workplace injuries, particularly needle-prick incidents among sewing operators. A worker described her experience: “I pricked my finger twice last week; it happens when we are too tired and sweaty.” Manual handling activities in high-temperature environments were also associated with fainting episodes and headaches, symptoms frequently experienced by employees across different factory departments. Moreover, access to drinking water was often limited, exacerbating dehydration and related symptoms.

In addition to physical issues, workers reported considerable mental health and cognitive challenges linked to excessive heat exposure. Commonly mentioned mental health effects included difficulty concentrating on tasks, reduced motivation, and feelings of irritability directed both at themselves and their colleagues. One participant succinctly described these emotional impacts, noting, “When it’s too hot, I get angry.” Such emotional responses highlight the profound influence that sustained heat exposure has on workers' psychological well-being and workplace interpersonal dynamics.

### Indoor air pollution

Table 4 highlights the substantial health impacts of air pollution on SMAMCs employees, particularly respiratory outcomes. Participants reported coughing 95.05% (95% CI; 92.4 to 96.8), respiratory issues symptoms 94.79% (95% CI; 92.1 to 96.6), allergies 94.01% (95% CI; 91.2 to 96.0), shortness of breath 70.31% (95% CI; 65.6 to 74.7), and eye diseases 70.57% (95% CI; 65.8 to 74.9). Pearson chi-square tests confirmed statistically substantial associations for shortness of breath (χ^2^ = 21.779, *p <* 0.001) and eye diseases (χ^2^ = 18.950, *p <* 0.001), with moderate Phi values of 0.238 and 0.222, respectively. Other symptoms, despite high prevalence, did not show statistical significance.

Qualitative responses, participants frequently reported respiratory health concerns directly associated with their work environment, often beginning shortly after joining the apparel industry. Commonly reported symptoms included wheezing, asthma-like conditions, and chronic breathing difficulties. One respondent clearly articulated this health impact, stating, “I never had asthma before, but now I wheeze almost every day after work.” Participants further emphasised that respiratory discomfort intensified on hot days due to increased air pollution from surrounding industrial activities. One worker described this scenario, stating, “When the heat is high, the outside smoke comes in, we can’t breathe properly and feel tired by noon.” These responses illustrate how industrial emissions, compounded by climatic conditions, significantly compromise respiratory health among apparel workers.

Workers consistently highlighted eye and skin-related health problems caused by poor indoor air quality, particularly in factories reliant on natural ventilation systems. Eye irritation, commonly referred to by workers as “sore eyes”, was frequently attributed to the influx of dust through open windows during the dry season. One participant noted, “The dust gets in through the windows, my eyes turn red and start feeling burning.” Workers also described allergic responses, including conjunctivitis and skin irritations, which reportedly worsened when handling particular fabrics under hot and dusty conditions. Another respondent remarked, “Some materials make my skin itch and turn red; it’s worse when it’s hot and the place is dusty.

### Mosquito-borne diseases

Study highlights a substantial prevalence of dengue at 10.68% (95% CI; 8.0 to 14.2) over the past five years, emphasising its extensive health implications. Statistical analysis with a Pearson chi-square result of r = 8.356, *p <* 0.003, shows a statistically significant link between Dengue and Environmental Factors. The conditions conducive to mosquito breeding, with a Phi value of 0.148, indicate a weak positive relationship.

Qualitative responses, participants consistently noted an increased presence of mosquitoes and insects during hot and dry periods, causing considerable discomfort and negatively impacting their ability to concentrate and maintain productivity at work. One participant explained, “In the hot season, the mosquitoes don’t let us work, they keep biting.” Such conditions often resulted in visible skin irritations, rashes, and bite marks on workers' limbs. Another worker remarked, “We get rashes and marks on our arms and legs after bites.” The frequent scratching and irritation from insect bites often led to secondary health complications, such as open wounds, increased risk of infection, and subsequent absenteeism. Employees expressed particular concern regarding these complications and the implications for their longer-term health. One participant described a specific incident, stating, “I had to see the doctor because the bite turned into a sore, and I had to take leave.”

## Discussion and conclusions

This study examined the multifaceted impacts of climate change-induced hazards flooding, excessive heat, indoor air pollution, and mosquito-borne diseases, on worker health, safety, and productivity within Sri Lanka’s small and medium apparel manufacturing companies (SMAMCs). The findings demonstrate direct, substantial, and cascading effects, highlighting a critical policy gap and emphasising the urgency of implementing integrated, climate-resilient workplace safety strategies.

**Fig. 2:**
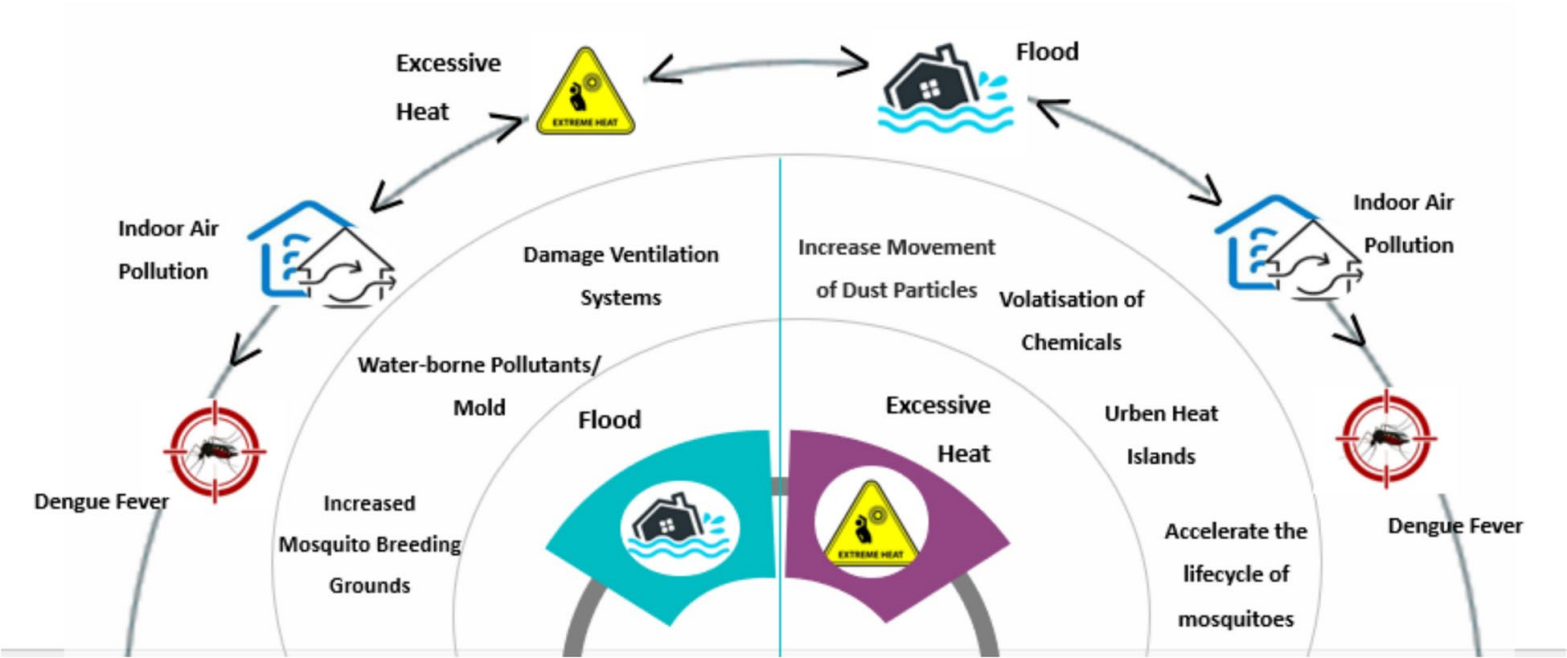
Interconnection of climate change-induced natural hazards (Source: Author)

Adverse weather, notably flooding, emerged as a significant threat to worker health, with respiratory issues (75.52%, 95% CI; 71.2 to 79.8), asthma (77.08%, 95% CI; 72.9 to 81.3), diarrhoea (47.40%, 95% CI; 42.4 to 52.4), and throat infections (81.25%, 95% CI; 77.3 to 85.2) prominently reported. These findings align closely with global research demonstrating that flooding exacerbates respiratory conditions due to mould proliferation and damp indoor environments [49, 50]. Additionally, psychological stress linked to extreme weather (29.95%, 95% CI; 25.3 to 34.6) resonates with broader studies that underline mental health risks following floods [51]. The frequent physical injuries and illnesses such as hepatitis, ringworm, and sore eyes) reported by respondents reflect the vulnerability of SMAMCs to infrastructure deficiencies and inadequate maintenance during floods, echoing findings by Saatchi et al. [52] in similar industrial contexts. Figure 2 illustrates both compounding effects where hazards such as flooding and excessive heat co-occur or occur in close sequence and interact to amplify risk and cascading effects, the chain of dependent impacts that begins with a climatic trigger, propagates through workplace pathways, and culminates in downstream outcome. The inner segment shows primary hazards; the middle ring shows workplace pathways; the outer ring shows consequences. Bidirectional arrows indicate potential compounding between flood and heat, while the ringed progression illustrates the cascade (See Fig. 2).

Heat exposure also posed significant health risks, notably excessive sweating (89.58%, 95% CI; 81.8 to 88.8), severe headaches (85.67%, 95% CI; 81.0 to 88.1), and dehydration (80.46%, 95% CI; 76.2 to 84.1), correlating closely with international evidence from tropical manufacturing environments, as reported by Moda et al. [53], ILO [50], and EU-OSHA [54]. These impacts are compounded by cognitive and psychological burdens such as reduced focus (85.41%, 95% CI; 81.5 to 88.6) and extreme fatigue (84.89%, 95% CI; 81.0 to 88.1), which significantly heighten occupational injury risks, confirming previous findings from the NIOSH [55] and EU-OSHA [54]. Limited workplace hydration access intensified these effects, underlining a crucial policy oversight requiring immediate intervention [10, 56].

Indoor air pollution was statistically associated only with shortness of breath and eye-related condition. Although coughing (95.05%, 95% CI; 92.4 to 96.8) and general respiratory discomfort (94.79%,95% CI; 92.1 to 96.6) were prevalent yet statistically inconclusive, eye diseases showed significant associations (70.57%, 95% CI; 65.8 to 74.9). These results are consistent with previous research linking particulate matter and industrial emissions to occupational eye and skin irritations [57, 58]. Respondents’ qualitative feedback specifically highlighted the exacerbation of respiratory and ocular symptoms on hot days, supporting existing studies that associate poor ventilation with deteriorating worker health in textiles [59, 60].

Mosquito-borne diseases, particularly dengue fever, were identified as a rising occupational hazard, correlating statistically with increased mosquito breeding conditions due to climatic variability. Dengue occurrences among workers substantiate earlier evidence highlighting climate-driven vector expansion [21, 61]. Outbreaks observed in industrially dense regions, such as Colombo and Kurunegala, reinforce the need for targeted environmental surveillance and proactive vector control measures [62, 63]. Additionally, the mental health implications associated with dengue and related infectious diseases align with findings from Gunathilaka et al. [63], reinforcing the importance of comprehensive, climate-sensitive occupational health strategies.

Importantly, hazards were not isolated. Flood damage to buildings increased mould exposure and impeded ventilation [64–66], compounding heat stress and dust inhalation [15]; heat, in turn, volatilised chemicals, aggravating respiratory symptoms [67]; and both flooding and heat drove mosquito abundance [68]. 

### Sri Lankan SMAMCs climate resilience action priorities

**Fig. 3:**
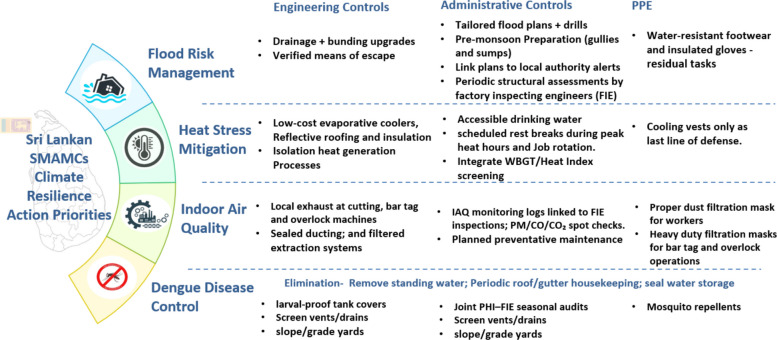
Sri Lankan SMAMCs climate resilience action priorities (Source: [50, 69, 70])

This study provides critical insights for SMAMCs *Sri Lankan SMAMCs climate resilience action priorities* (see Fig. 3).

Recommendations are structured by the risk-control hierarchy, with compliance grounded in the Factories Ordinance Cap. 128 provisions on drainage of floors, cleanliness, ventilation, temperature control, safe egress, and medical supervision, the Disaster Management Act No. 13 of 2005, and the National Disaster Management Plan 2023–2030 [69–71].

Engineering priorities are retrofitting floor drainage and bunding, elevating electrical isolation above flood lines and verifying means of escape through auditable testing. Administrative priorities are site-specific flood plans and drills aligned with national disaster frameworks, pre-monsoon housekeeping of gullies and sumps, contractor controls, and documented follow-up to Factory Inspecting Engineer inspections [72]. These measures are consistent with international guidance, with PPE reserved as residual protection only [54, 55, 73].

The Factories Ordinance provides powers on ventilation, thermal conditions and medical supervision, and the National Occupational Safety and Health Policy call for standards, surveillance and employer duties [71, 72]. Preferred controls are engineering measures such as low-cost evaporative coolers, reflective or high-albedo roofing, added insulation, and isolating heat‑generating processes, such as heat‑seal machines and pad printing. Administrative measures include heat-health surveillance, acclimatisation protocols, task or job rotation away from peak‑heat periods, and routine use of Wet Bulb Globe Temperature or Heat Index thresholds in inspections and work–rest planning, consistent with NIOSH criteria and current EU-OSHA guidance [54, 55]. PPE such as cooling vests is retained as the final barrier.

Legal anchors are the Factories Ordinance on adequate ventilation and the rendering harmless of injurious fumes and dust, and the National Environmental Ambient Air Quality Regulations under the National Environmental Act [71, 74]. Elimination or substitution includes lower-dust inputs where practicable. Engineering measures prioritise local exhaust ventilation at bar‑tag and overlock machines, sealed ducting with filtered extraction, and periodic measurement against national ambient or area benchmarks to trigger corrective actions [54, 75]. Administrative controls cover indoor air quality logs tied to inspections, planned preventive maintenance of ventilation systems, and targeted worker training. Respiratory PPE is retained as a last line of defence, with routine dust-filtration respirators for general tasks and higher-filtration respirators for bar‑tag and overlock operations where engineering and administrative controls do not reduce exposure adequately [54, 76].

Compliance is anchored in the Prevention of Mosquito Breeding Act No. 11 of 2007, which imposes duties on owners and occupiers to eliminate breeding sites, enforced by Public Health Inspectors and supported by national dengue control guidance and global alerts [77–80]. Elimination emphasises removal of standing water, routine roof and gutter housekeeping and sealing of water storage. Engineering measures include screened vents and drains and larval‑proof tank covers. Administrative measures comprise joint Public Health Inspector–Factory Inspecting Engineer seasonal audits around drought and monsoon onset, worker reporting protocols and contractor controls for landscaping and waste. Topical repellents are retained as residual PPE only. This auditable interface operationalises statutory public‑health duties in factory practice.

The cross-sectional design restricts causal inference and the tracking of health outcomes over time. Although the sample was statistically robust, recall bias and self‑reporting constraints may influence estimates. Nonetheless, triangulation with qualitative evidence and statistically supported associations across several health dimensions provide a substantial basis for inference. Insights are most transferable to similarly structured low‑ and middle‑income apparel sectors with comparable climatic and infrastructural profiles, and caution is advised when extrapolating beyond such contexts.

Longitudinal designs are needed to track climate‑related occupational health trends and to assess policy and programme effectiveness. Cost–benefit analyses of engineering controls, including improved ventilation or local exhaust ventilation, flood‑proofing and cooling technologies, would strengthen the business case for resilience investments. Cross‑national comparisons could elucidate regional variation in regulatory performance and adaptation pathways, enabling best‑practice transfer. Mixed‑methods designs that integrate qualitative assessments with quantitative modelling can better capture worker perceptions and organisational behaviour, supporting interventions that are both scientifically robust and socially acceptable.

## Supplementary Information

Supplementary Material 1.

## Data Availability

The data that support the findings of this study are available from the corresponding author (DS), upon reasonable request.
