# Supplementary material for: Workplace health and safety under climate stress in Sri Lankan apparel SMEs
Source: BMC Public Health. 2026 Jan 20;26:587. doi: 10.1186/s12889-026-26344-1 (PMC12903649; doi:10.1186/s12889-026-26344-1)
Supplement: Supplementary file 1 — Supplementary Material 1. [file 12889_2026_26344_MOESM1_ESM.docx]

| Please tick your chosen answer. | YES | NO |
| --- | --- | --- |
| I have read the information and have had the opportunity to ask questions. | ☐ | ☐ |
| I am giving my consent to take part in the survey. | ☐ | ☐ |

1. What is your current designation?

| Team Member | Senior Team Member | Multiskilled operator | Assistant Line Leader | Line Leader |
| --- | --- | --- | --- | --- |
|  |  |  |  |  |

1. What is the annual turnover of your company?

| Less than Rs.10 million | Rs.10 million to Rs.24.99 million | Rs.. 25 million to Rs.49.99 million | Rs.50 million to Rs.74.99 million | Above Rs.75 million | Don’t know |
| --- | --- | --- | --- | --- | --- |
|  |  |  |  |  |  |

1. What is the size of the workforce in your company?

| Less than 10 workers | 10 to 50 workers | 51 to 100 workers | 101 to 150 workers | More than 150 workers | Don’t know |
| --- | --- | --- | --- | --- | --- |
|  |  |  |  |  |  |

1. How long have you worked in the current company?

| Less than 1 year | 2 to 5 years | 5.1 to 10 years | 10.1 to 15 years | More than 15 years |
| --- | --- | --- | --- | --- |
|  |  |  |  |  |

1. How long have you worked in the Sri Lankan Apparel manufacturing industry?

| Less than 1 year | 2 to 5 years | 5.1 to 10 years | 10.1 to 15 years | More than 15 years |
| --- | --- | --- | --- | --- |
|  |  |  |  |  |

1. Does your company have safety guidelines?

| Yes | No | Don’t know |
| --- | --- | --- |
|  |  |  |

1. As you know, how long has your company followed occupational health and safety guidelines?

| Less than 1 year | 1 to 5 years | 5.1 to 10 years | 10.1 to 15 years | More than 15 years | Don’t know |
| --- | --- | --- | --- | --- | --- |
|  |  |  |  |  |  |

1. Do your company's current health and safety guidelines include measures to reduce the effects of bad weather events such as flooding, storms, and high temperatures?

| Yes | No | Don’t know |
| --- | --- | --- |
|  |  |  |

1. Which climate change-related weather events can harm the health and safety of our company's employees? (You can select all that are relevant)

| Adverse weather events (flooding, cyclones, earth-slips, lightning, extreme wind) | Heat waves | Air  pollution | High exposure to sunlight | The risk of contagious diseases spread by mosquitoes, ticks, or fleas | Other (Please specify) |
| --- | --- | --- | --- | --- | --- |
|  |  |  |  |  |  |

…………………………………………………………………………………………………………………………………………………………………………………………………………………………………………………………………………………………………………

1. In the past five years, what are the main illnesses and injuries that you and other workers in your company have experienced due to increased extreme rainfall, lightning, and flooding? (Select all that are relevant)

| Less motivation |  | Mosquito Bites |  |
| --- | --- | --- | --- |
| Less focus |  | Sinus |  |
| Respiratory Issues |  | Sore throat and other throat irritations |  |
| Electrocution and electrocution risks |  | Stress |  |
| Asthma |  | Cold and fever |  |
| Diarrhoea |  | Anxiety |  |
| Drowning due to flooding |  | Depression |  |
|  |  | Other (Please specify) |  |

……………………………………………………………………………………………………………………………………………………………………………………………………………………………………………………………………………………………………………………

1. In the last five years, what are the main illnesses and injuries that you and other workers in your company have experienced due to the rise in average temperatures in the country? (Select all that are relevant)

| Excess sweating |  | Body Pains |  |
| --- | --- | --- | --- |
| Headache |  | Kidney diseases |  |
| Fainting |  | Dehydration |  |
| Heart pains |  | Reduced attention |  |
| Heat exhaustion |  | Extreme fatigue |  |
| Heat rash |  | Occupational Hazards |  |
| Heat stroke |  | Other (please specify) |  |

……………………………………………………………………………………………………………………………………………..

……………………………………………………………………………………………………………………………………………………

1. In the past five years, what are the main illnesses and injuries that you and other workers in your company have experienced due to the increase in dust particles, carbon dioxide, and other pollutants in the air? (Select all that are relevant)

| Coughing |  | Dust Allergies |  |
| --- | --- | --- | --- |
| Shortness of breath |  | Eye diseases |  |
| Respiratory Issues |  | Other (Please specify) |  |
| Heart pain |  |  |  |

…………………………………………………………………………………………………………………………………………………………………………………………………………………………………………………………………………………………………………

1. and injuries that have affected you and other workers in your company due to the increased exposure to bright sunlight over the last five years? (Select all that are relevant)

| Skin darkening |  | Dry skin |  |
| --- | --- | --- | --- |
| Skin rashes |  | Other (Please specify) |  |
| Skin cancer |  |  |  |

1. To your knowledge what are the main illnesses that have affected you and other workers in your company due to the increase in the population of malaria, dengue, and other disease-carrying mosquitoes over the last five years? (Select all that are relevant)

| Dengue |  | Zika Virus |  |
| --- | --- | --- | --- |
| Malaria |  | West Nile Virus |  |
| Chikungunya Fever |  | Other (Please specify) |  |

1. Have you noticed any efforts in your workplace to reduce its environmental pollution?

| Solar panel systems |  |
| --- | --- |
| water cooling systems |  |
| Staff transport provided |  |
| Staff accommodation within walking distance |  |
| Cycle allowances |  |
| Water treatment plant |  |
| Waste recycling |  |
| Combined staff transport services for Holidays |  |
| Outdoor Cleaning Schedules |  |
| DFI/PHI visits |  |
| Usage of LED bulbs |  |

Other ……………………………………………………………………………………………………………………………………

1. What systems are used at work to remove dust and harmful particles from the air?

| Dust Extraction System |  |
| --- | --- |
| Dust Exhaust System |  |
| Air Purifier system |  |
| Dust collecting system |  |

Other ……………………………………………………………………………………………………………………………..

1. What changes are made by owners in building design and construction to protect workers from climate change-related hazards?

| Built flood protection walls |  |
| --- | --- |
| Heat buffer walls made with bricks |  |
| Walls are insulated |  |
| Maintaining a proper drainage system |  |
| Good Air circulation with windows |  |
| Layout changes in monsoon periods accordingly |  |
| Built fire protection walls |  |

Other ……………………………………………………………………………………………………………………………..

1. What are the systems/tools used by the company to monitor the health and safety environment in the workplace?

| Software systems |  |
| --- | --- |
| Internal Auditing |  |
| Checklists |  |
| External Customer Audits |  |
| Internal Management Systems |  |
| Management Systems (ISO 14001/45001) |  |

Other ……………………………………………………………………………………………………………………………..

1. What regulations and policies have the company implemented to protect workers' health and safety from climate change-related hazards? ­

| Sri Lankan Factories Ordinance - |  |
| --- | --- |
| ILO guidelines |  |
| International guidelines and policies |  |
| Policies by Brands |  |
| Policies by local Bodies (Central Environment Authority, Local Municipality Councils) |  |

Other ……………………………………………………………………………………………………………………………..

Please answer questions 15 to 19 by selecting an option from the below table 01.

Table 01

| **1** | **2** | **3** | **4** | **5** |
| --- | --- | --- | --- | --- |
| No illnesses/injuries to employees. | Illnesses/injuries to less than 5 employees. | Illnesses/injuries to 5 to 10 employees. | Illnesses/injuries 10-15 employees/, 1-5 hospitalised. | Illnesses/injuries to more than 15 employees/Over 5 Hospitalised |

Aware of the data

| Yes | No | Don’t know |
| --- | --- | --- |
|  |  |  |

If your answer is **Yes** answer 16 – 20 or Move to question no 21

1. Using the scales shown in Table 1 please indicate the illnesses/injuries in your company due to the increase in extreme rainfall, flooding, and lightning over the last two years.

| 1 | 2 | 3 | 4 | 5 |
| --- | --- | --- | --- | --- |
|  |  |  |  |  |

1. Using the scales shown in Table 1 please indicate the illnesses/injuries to workers in your company due to the increase in the average temperatures of the company over the last two years?

| 1 | 2 | 3 | 4 | 5 |
| --- | --- | --- | --- | --- |
|  |  |  |  |  |

1. Using the scales shown in Table 1 please indicate the illnesses/injuries to workers in your due to the increase in dust particles, carbon dioxide and other pollutants in the air over the last two years?

| 1 | 2 | 3 | 4 | 5 |
| --- | --- | --- | --- | --- |
|  |  |  |  |  |

1. Using the scales shown in Table 1 please indicate the illnesses/injuries to workers in your company due to the increased exposure to bright sunlight over the last two years?

| 1 | 2 | 3 | 4 | 5 |
| --- | --- | --- | --- | --- |
|  |  |  |  |  |

1. Using the scales shown in Table 1 please indicate the illnesses/injuries in your company due to the increase in the population of malaria, dengue, and other disease-carrying mosquitoes over the last two years?

| 1 | 2 | 3 | 4 | 5 |
| --- | --- | --- | --- | --- |
|  |  |  |  |  |

1. What safety measures does your workplace have in place to protect employees from adverse weather events? (Select all that are relevant)

| Reduce exposure |  |
| --- | --- |
| Introduce different techniques/procedures. |  |
| Physical developments and engineering controls |  |
| A safe system of work – Instructions/guidelines |  |

1. What safety measures does your workplace have in place to protect employees from excessive Heat? (Select all that are relevant)

| Reduce exposure |  |
| --- | --- |
| Introduce different techniques/procedures. |  |
| Physical developments and engineering controls |  |
| A safe system of work – Instructions/guidelines |  |

1. What safety measures does your workplace have in place to protect employees from air pollution? (Select all that are relevant)

| Reduce exposure |  |
| --- | --- |
| Introduce different techniques/procedures. |  |
| Physical developments and engineering controls |  |
| A safe system of work – Instructions/guidelines |  |
| Wear mask |  |

1. What safety measures does your workplace have in place to protect employees from high exposure to sunlight? (Select all that are relevant)

| Reduce exposure |  |
| --- | --- |
| Introduce different techniques/procedures. |  |
| Physical developments and engineering controls |  |
| A safe system of work – Instructions/guidelines |  |

1. What safety measures does your workplace have in place to protect employees from mosquito-borne diseases? (Select all that are relevant)

| Reduce exposure |  |
| --- | --- |
| Introduce different techniques/procedures. |  |
| Physical developments and engineering controls |  |
| A safe system of work – Instructions/guidelines |  |

1. What measures have been implemented by the owners of your workplace to reduce the impact of climate change events on Occupational Health and Safety? ( Select all that are relevant)

| Education/Training |  | Use of appropriate PPEs |  |
| --- | --- | --- | --- |
| Introduction of work and rest cycles |  | Medical monitoring |  |
| Introduction of new health and safety policies and regulations |  | Other (Please specify) |  |

1. What steps do you take to reduce the impact of climate change on Occupational Health and Safety?

| Requesting Rest Periods |  | Medical monitoring |  |
| --- | --- | --- | --- |
| Wearing PPE |  | Ensuring appropriate work and rest periods |  |
| Ensuring hydration |  | Other (Please specify) |  |

Other ……………………………………………………………………………………………………………………………..

1. What engineering or mechanical measures have you observed in your workplace to reduce the impact of climate change on Occupational Health and Safety? ( Select all that are relevant)

| Use of cooling/heating systems |  |
| --- | --- |
| Focus on reducing greenhouse gases. |  |
| Systems to extract dust and other particles from the air |  |
| Modification/construction of buildings to reduce the threat of hazards caused by climate change |  |
| Monitoring of office health and safety environment using various systems |  |
| Health and safety regulations are introduced by the industry and state |  |
| Other (Please specify) |  |

Other ……………………………………………………………………………………………………………………………..

1. To what extent have measures introduced by owners' controls have prevented or reduced illnesses/injuries to workers in your company from climate change-related adverse weather events?

| Extremely ineffective (Over 50% of workforce has been impacted by diseases/illnesses caused by climate change) | Ineffective (30% to 50% of the workforce has been impacted by diseases/illness caused by climate change | Somewhat effective (between 10% to 29%. of workforce has been impacted by climate change related diseases | Effective (between 1% to 10% of the workforce has been impacted b diseases/illnesses caused by climate change. | Extermely effective (No workers have been impacted by illnesses and diseases caused by climate change) |
| --- | --- | --- | --- | --- |
|  |  |  |  |  |

Other ……………………………………………………………………………………………………………………………..

1. To what extent do you think your own measures prevented or reduced illnesses/injuries to you from climate change-related adverse weather events?

| Extremely ineffective (Over 20 days of medical leave taken in the last two years due to climate change related illnesses/diseases) | Ineffective (15 to 20 days of medical leave taken during the last two years for climate change related illnesses/diseases) | Somewhat effective (10 to 14 days of medical leave taken during the last two years for climate change related illnesses/diseases) | Effective (5 to 9 days of medical leave taken during the last two years for climate change related illnesses/diseases) | Extremely effective (Less than 6 days of medical leave taken during the last two years for climate change related illnesses/diseases) |
| --- | --- | --- | --- | --- |
|  |  |  |  |  |

Other ……………………………………………………………………………………………………………………………..

1. Has your company allocated resources to identify new hazards emerging due to climate change?

| Yes | No | Don’t know |
| --- | --- | --- |
|  |  |  |

1. Do you participate in annual worker health and safety exposure assessments at your workplace?

| Yes | No | Don’t know |
| --- | --- | --- |
|  |  |  |

1. To what extent do you believe your workplace implemented measures to control and mitigate Occupational Safety risks to workers arising from climate change hazards?

| A controlled approach has not been adopted | A controlled approach has been adopted to a certain extent | 100% adoption of a controlled approach | Don’t know |
| --- | --- | --- | --- |
|  |  |  |  |

Other ……………………………………………………………………………………………………………………………..

1. In addition to measures taken by owners, what are the personal safety controls followed by you and/or other workers in your factory to prevent and reduce illnesses/injuries arising from the increase in adverse weather events caused by climate change?

| Always keep a water bottle to reduce dehydration. |  |
| --- | --- |
| Consume the break time in full at the Cafeteria |  |
| Wearing a mask is irrespective of the machine that operates. |  |
| Having short breaks while operating with high cotton dust generation sewing machines |  |
| Inform supervisors to cover up the roof lanterns. |  |
| Always be at a shelter or the cafeteria till company transport arrives. |  |
| Use a clean, wet cloth to keep on the neck during high-temperature days |  |

Other …………………………………………………………………………………………………………………………………….

1. What are the education and training programs that you have participated in to get aware of reducing health and safety risks from adverse weather events and other hazards caused by climate change?

| Safety induction |  |  |  |  |  |
| --- | --- | --- | --- | --- | --- |
| Bi-annual safety refresher awareness |  |  |  |  |  |
| Occupational First-Aid Training |  |  |  |  |  |
| Occupational firefighting and rescue training |  |  |  |  |  |
| Chemical handling training |  |  |  |  |  |
| Safety day awareness |  |  |  |  |  |
| Training conducted in the first week of June (Environment Day celebration) |  |  |  |  |  |
| Environment Day activities |  |  |  |  |  |
| Training conducted by the Safety, Health and Environment Committee |  |  |  |  |  |

Other ……………………………………………………………………………………………………………………………………………....

1. What are the work and rest cycles that you follow to reduce Occupational Health and Safety risks from hazards caused by climate change?

…………………………………………………………………………………………………………………………………………………………..

1. Please describe any personal health and safety suggestions/ideas given by you and/or other workers in your factory to prevent and reduce illnesses/injuries due to the increase in adverse weather events caused by climate change.

…………………………………………………………………………………………………………………………………………………………………………………………………………………………………………………………………………………………………………………...

1. What is the Personal Protective Equipment (PPE) requested by you and/or other workers to mitigate the threat to Occupational Health and Safety from hazards caused by climate change?

| Face Masks |  |
| --- | --- |
| Anti-fatigue carpets |  |
| Heat Resistant gloves |  |
| Face shields |  |
| Spectacles |  |
| Anti slippery grips |  |
| Safety Shoes |  |
| Earplugs |  |
| Hair Nets |  |
| Fully Cotton workwear |  |

Other …………………………………………………………………………………………………………………………………….

1. Please describe any occupational medical monitoring requested by you and/or other workers in your factory from owners to prevent and reduce illnesses/injuries from the increase in adverse weather events caused by climate change.

| Lung Function test |  |
| --- | --- |
| Hearing test |  |
| Palm culture test |  |
| Skin test |  |
| Vision test |  |
| sputum culture (Cough) culture test |  |
| Skin culture test |  |

Other …………………………………………………………………………………………………………………………………….

1. What are the personal practices followed by your and/or other workers in hot days in your factory to prevent or reduce illnesses/injuries from the increase in adverse weather events?

………………………………………………………………………………………………………………………………………………………………………………………………………………………………………………………………………………………………………
